# Methods of experimental study of thermal convection in cavity subject to rotation and vibration

**DOI:** 10.1016/j.mex.2019.10.005

**Published:** 2019-10-11

**Authors:** Viktor G. Kozlov, Alexei A. Vjatkin, Rustam R. Sabirov, Vladimir M. Myznikov

**Affiliations:** a Perm State Humanitarian Pedagogical University, Perm, Russia; b Perm National Research Polytechnic University, Perm, Russia

**Keywords:** The method of experimental study of vibrational thermal convection, Vibrational thermal convection, PIV-method, Rotation, Vibration, Heat transport enhancement

## Abstract

Vibrations of a non-isothermal fluid result in the generation of an averaged flows – “thermal vibrational convection”. The effect of vibrations changes qualitatively, and their effectiveness increases significantly under conditions of rotation. This makes vibrations an important tool for controlling the dynamics of non-isothermal systems and the intensification of heat and mass transfer in rotating cavities, which could be useful for optimization of various technological processes. The article describes methods used to study convection in rotating cylinders subject to vibrations. The cases of an annulus with boundaries at different temperatures and a cylinder filled with a heat-generating fluid are discussed. Experimental complex allows one to study thermal convection under the action of various factors, such as rotation, vibrations, librations, orientation of the axis of rotation, inertial waves, and others.

• The vibrations of a non-isothermal fluid in a rotating cavity is an effective tool for intensification of heat transport due to the generation of intensive averaged flows
• The developed experimental technique and setup provide the investigation of thermal vibrational convection in rotating cavities of various geometries in a wide range of governing dimensionless parameters

## Graphical abstract

**Figure:**
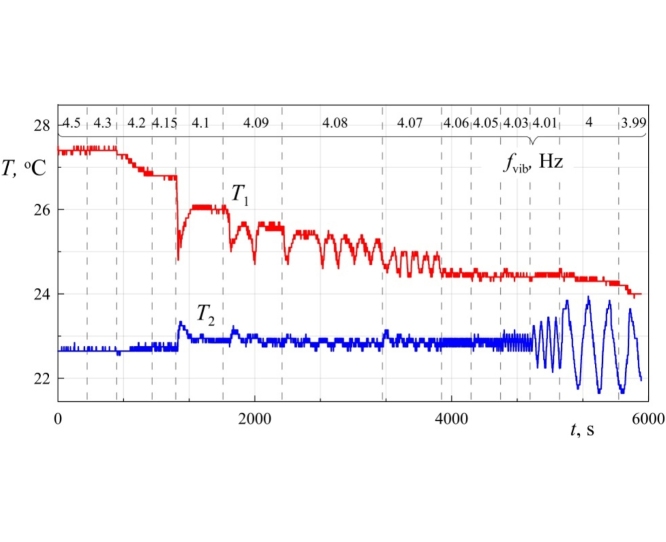

**Specification Table**

**Table:** 

Subject Area:	Physics and Astronomy
More specific subject area:	Thermal convection of fluid in oscillating force fields
Method name:	The method of experimental study of vibrational thermal convection
Name and reference of original method:	If applicable, include full bibliographic details of the main reference(s) describing the original method from which the new method was derived.
Resource availability:	If applicable, include links to resources necessary to reproduce the method (e.g. data, software, hardware, reagent)

## Method details

### Introduction

Vibrations are an effective tool for controlling thermal convection and heat transfer in a non-isothermal fluid. Under the action of high-frequency translational vibrations, the non-isothermal fluid makes forced oscillations in an oscillating inertial force field, which results in generation of an averaged lift force. "Thermal vibrational convection" [1], which manifests itself in this case, does not depend on static force fields and can be effectively used to induce convection under conditions of low gravity or to suppress the natural convection in a gravity field.

Significant changes in the mechanism of vibrational thermal convection are observed under the vibration of a rotating cavity with a non-isothermal fluid. Experimental studies [2,3] and the theoretical description of vibrational convection [4] correspond to the case of high-frequency oscillations of a force field in the reference frame of a rotating cavity. In this case, convection is determined by a specific mechanism and new dimensionless parameters.

Qualitative differences in the action of vibrations on a rotating non-isothermal fluid were found when the rotation frequency is close to the frequency of vibrations [5]. It is shown that when the frequencies coincide, the action of vibrations is reduced to the generation of a high-intensity static convection in the cavity reference frame. Thus, vibrations can be used for operational external control of heat transfer in rotating cavities. The control can be carried out by changing both the amplitude and the relative frequency of vibrations. This work is devoted to the description of this method.

### Experimental technique

**Fig. 1:**
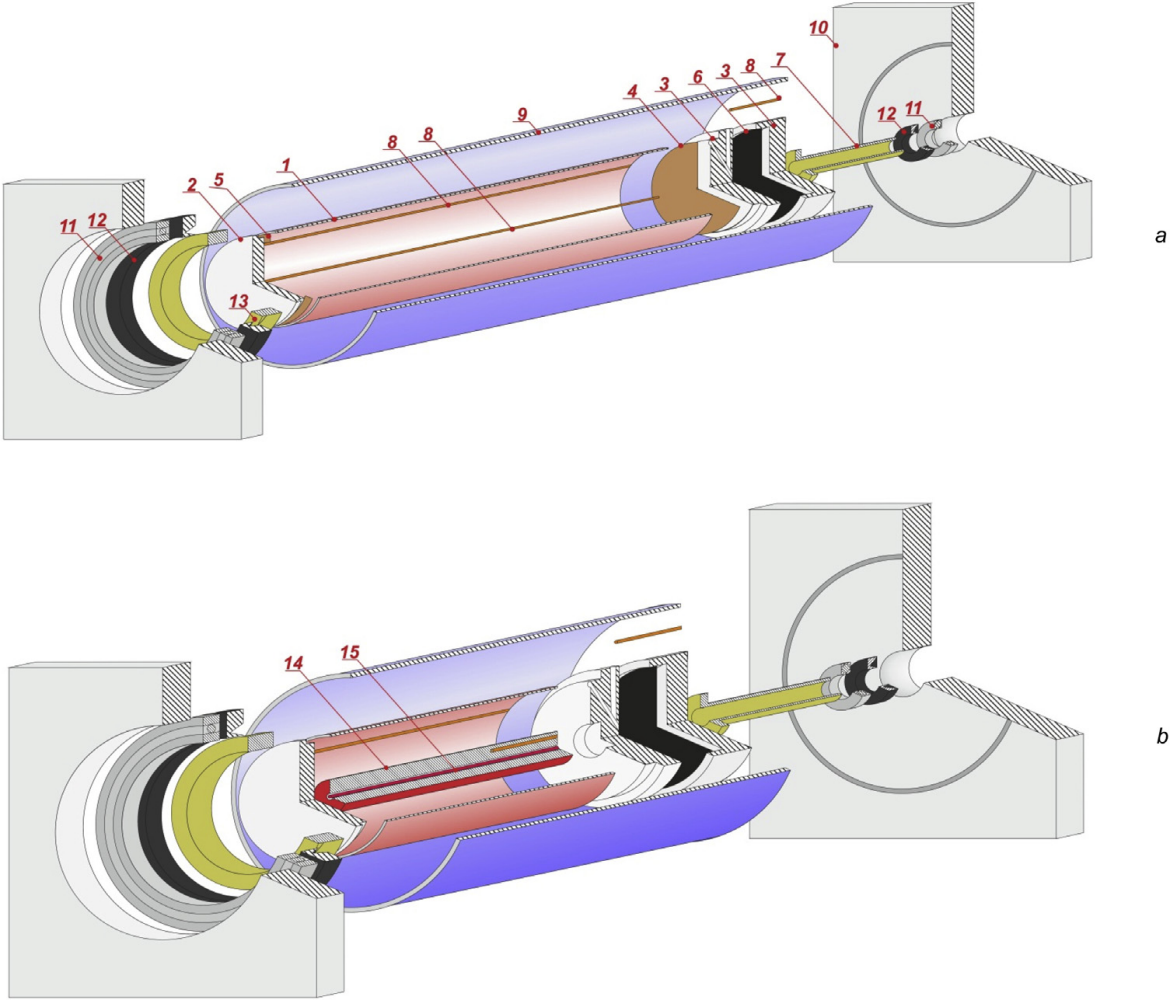
Schemes of the cuvettes for studying the convection of a heat-generating fluid in a rotating cylinder (a) and the convection in a rotating cylindrical fluid layer heated from the inside (b).

To study the thermal vibrational convection in rotating cavities two experimental devices were manufactured. The first of them is used to study the convection of heat-generating fluid in a rotating cylinder (Fig. 1а), and the second one – convection in a cylindrical fluid layer with boundaries of different temperatures (Fig. 1b). In the first case the cavity is formed by a Plexiglas tube 1 (Fig. 1а) with a wall thickness equals 3 mm, length l= 220 mm and inner diameter d= 44 mm. One end of the tube is closed by a transparent Plexiglas flange 2, which gives the opportunity to observe the behavior of the fluid. The second flange 3 is made up of two plastic parts, and serves to transmit the rotation of the cylindrical cavity, switching temperature sensors with measuring equipment and filling the cavity with working fluid. Heat generation in liquid is ensured by passing an alternating electric current. To improve the conductivity a small (up to 5% by weight) amount of copper sulfate is added in the liquid. Copper electrodes 4 and 5 are attached to the flanges from the inside. Electrode 4 is a disc with the thickness 1 mm. It occupies the entire area of the flange 3. Electrode 5 is made in the form of a narrow ring for visual observation through the flange 2. Alternating current makes it possible to eliminate difficulties with gradients of chemical potentials arising under the action of the electric field and consumption of a substance of electrodes during the experiment. In a cell with copper electrodes filled with a solution of copper sulfate, there is no problem of chemical transformations, which can lead to changes in the properties of the liquid. The variable potential difference causes ions to oscillate around a certain average position, rather than moving from one electrode to another, as it occurs in the case of direct current. An AC source GW Instek APS-9501 is used in the experiments. This device provides alternating current changing according to the harmonic law with the frequency 50 Hz, output voltage instability not more than 0.1 V and signal distortion factor no more than 0.5%. The current source is equipped with voltage, frequency, current and power meters. Power measurement error does not exceed 1%.

Flange 3 (Fig. 1a) is made up of two parts with technological channels serving for switching sensors and electrodes with measuring equipment and an alternating current source. Filling the cuvette with the working solution is also carried out through the channels in the flange. The working fluid contacts with the cavity formed when the parts of the flange are connected. An elastic membrane 6 is installed inside the cavity in order to compensate the excess pressure of the liquid, appearing as a result of thermal expansion during the passage of electric current. During the process of filling the cuvette with a working solution, the absence of gas and solid inclusions in all channels and cavities is strictly controlled. The parts of the flange are connected coaxially and hermetically. A bronze shaft 7 with through hole for leading wires is attached to the flange outside. The shaft is driven by a stepper motor FL86STH156, which is controlled by the driver SMD-78. Rotational velocity ranges from 0.01 to 5.00 rps and is set by ZETLab digital generator signals. The instability of the rotational velocity does not exceed 0.01 rps.

The temperature is measured by thermoresistors. With the development of convective flows, local temperature distributions appear in the cavity. In this case, the data of the point temperature sensor may depend on its location. To eliminate this effect, temperature measurements are carried out with integrated sensors, which show temperatures averaged over the entire cavity length. The arrangement of thermoresistors in the case of a heat-generating fluid is shown at Fig. 1(a, 8). Sensor T_1_, located on the axis of rotation of the cylinder is a few loops of copper wire with a diameter 0.02 mm, elongated along the entire length of the cavity and placed in a glass capillary with a diameter 2.5 мм. Sensor T_2_ on the cylindrical wall of the cavity is also made of several loops of copper wire with a diameter 0.02 mm, glued to the cylinder with a thin self-adhesive film with a thickness 0.1 мм. The resistance of sensors equals 100 ohms.

A temperature at the outer edge of the working cavity is kept constant. For this purpose the cuvette is placed in a Plexiglas tube 9 (Fig. 1a) of higher diameter, closed with flanges 10. Water of a predetermined temperature from the jet thermostat is pumped in the gap formed between the tubes. The consistency and uniformity of coolant temperature is monitored by sensor T_3_, made of thin copper wire wound on a non-conductive core. This sensor is placed in sealed enclosures and installed in the coolant flow near the outer surface of the working cavity. The system of bearing 11 and seals 12 allows the working cavity to rotate freely, while the outer tube 9 remains fixed. The cavity on the side of the viewing end is fixed inside the bronze bushing 13. Outside the bushings the bearing and seal are installed. The construction is hermetical, coaxial and provides the possibility to monitor the movement of fluid.

Another experimental setup, which allows studying the behavior of a fluid in the cylindrical layers (Fig. 1b), has a similar construction. An exception is the method of heating the liquid. An aluminum cylinder 14 with the diameter 22 mm is mounted on the axis of the cavity. On the axis of the cylinder there is a through hole for installing an electric heater 15. The high thermal conductivity of aluminum ensures uniform temperature at the inner boundary of the layer. To protect against corrosion and increase contrast when observing, the external surface of the aluminum cylinder, in contact with the liquid, is covered with a black self-adhesive film of thickness 0.1 mm. The heater is powered by direct current. The inner radius of the cylindrical layer is 11 mm, the outer one is 37 mm and the layer length is 185 mm.

**Fig. 2:**
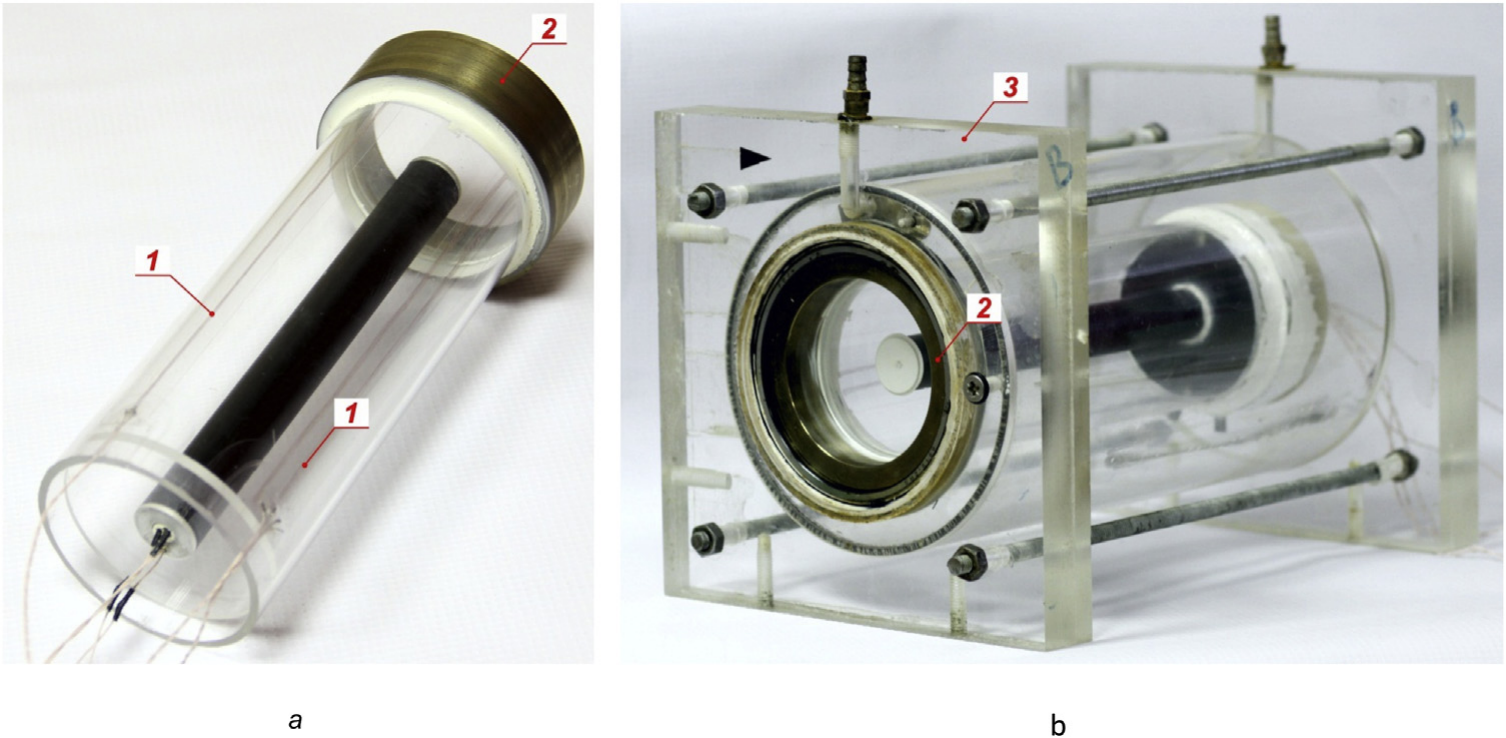
Photo of the working cavity (a) and the assembled cuvette (b).

Photo of the working cavity is presented at Fig. 2(a). Sensors 1, which measure temperature T_2_ are glued to the Plexiglas cylinder The copper wires of the sensors are connected by soldering to the lead wires on the outer edge of the cylinder. The junction place is sealed securely from the coolant. A bronze bushing 2 is installed on the side of the transparent flange to fit the working cavity into the outer transparent casing 3 (Fig. 2b).

**Fig. 3:**
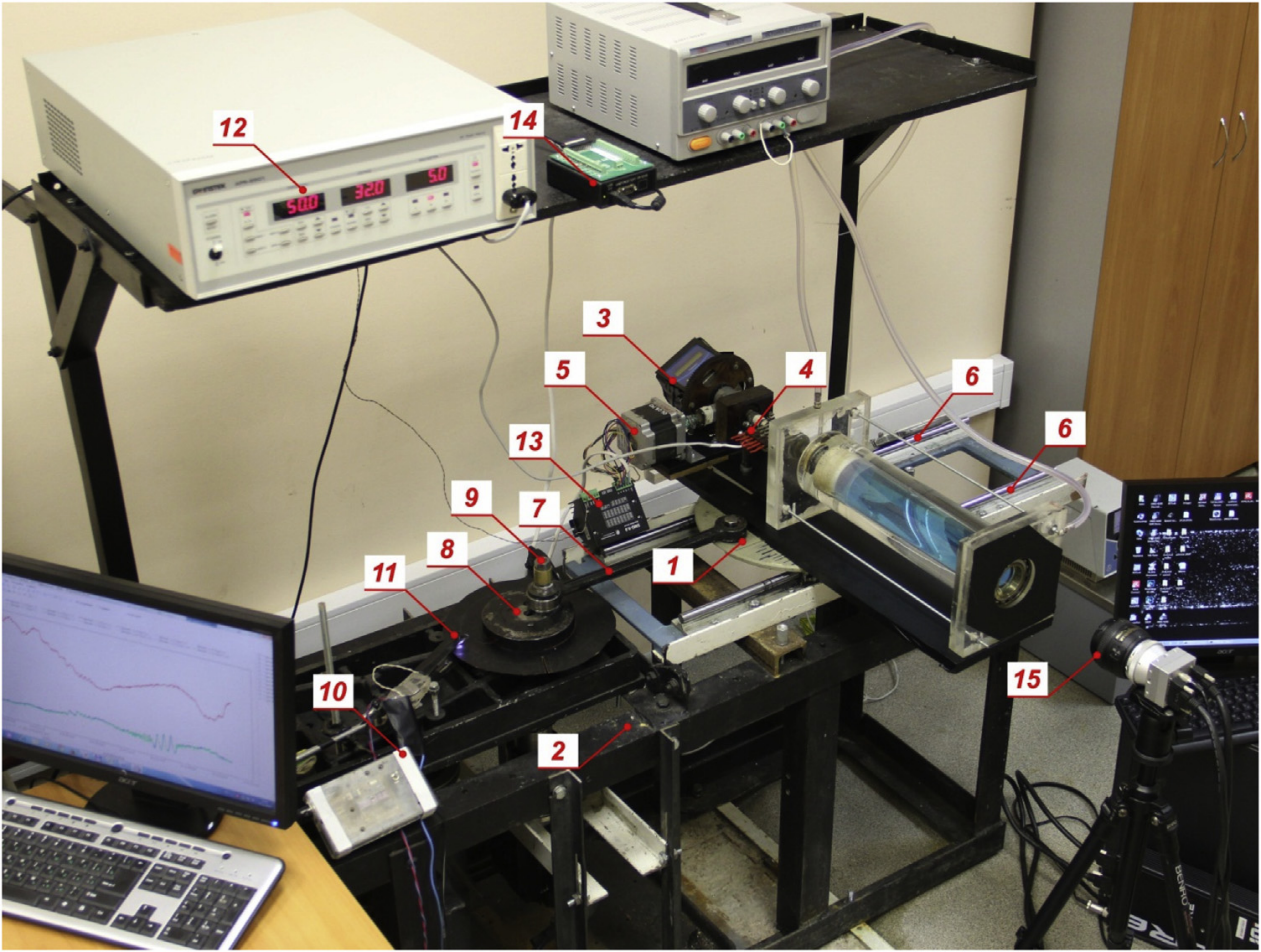
General view of the experimental complex.

The cuvettes described above are mounted on a horizontal desk 1 (Fig. 3) of mechanical shaker 2, setting the translational vibrations in a wide range of frequencies and amplitudes. A temperature meter "Termodat" 3 rotates with the cavity. This ensures reliable electrical contact between the temperature sensors and the measuring device. For electric power supply "Termodat" and connecting it to a PC, a multichannel electric collector 4 with sliding contacts is used. The stepper motor 5 through a bevel gear sets the rotation. The voltage on the copper electrodes attached to the inner sides of the flanges of the working cavity is also supplied using a collector 4. The vibrator table 1 moves along the rails 6 with the help of linear motion bearings. The translational vibrations of the desk are set by a crank mechanism, consisting of a connecting rod 7 with a length of 35 cm and a disk 8 with an eccentric axis 9. The amplitude of the vibration is determined by the radial displacement of the axis 9 relative to the center of the disk 8. The rotation of the disk 8 is transmitted from the servomotor ESTUN EMG-15APA22 with an amplifier PRONET-E-15A, ensuring the maintenance of frequency of vibrations with an accuracy not less than 0.2%. The frequency of vibrations is measured using a digital tachometer 10, connected to an optoelectronic pair 11. An optical cathetometer В-630 with accuracy 0.1 mm is used to measure the amplitude of vibrations in a range b = 0.1–30.0 mm. The photo also shows the AC source 12, the driver of the stepper motor 13, the digital signal generator 14 and the high-speed video camera 15.

In experiments with the visualization of flows a high-speed video camera CamRecord CL600 × 2 15 (Fig. 3) is set so that its optical axis is parallel to the axis of rotation of the cuvette. Shooting is carried out with a frequency multiple to the frequencies of rotation or vibrations. The flows are visualized using plastic light-scattering particles with a diameter of 60 μm and a density of about 1100 kg/m^3^, which are added to the working solution in an amount of not more than 0.1% by weight. Particles are suitable both for taking photos and for studying flows using the PIV-method [6]. Shooting is carried out in the plane of the light sheet, created by Z-Laser Z500Q.

### Experimental procedure and method validation

Before the experiment begins, a relatively low temperature T_3_ (19–22 °C) on the outer boundary of the cylinder is set and maintained constant using a thermostat. After a constant uniform temperature is established in the cavity, calibration of the temperature sensors is performed. Next, the cuvette is driven at a relatively fast (up to 4 rps) rotation, and an alternating voltage is applied to the electrodes. The value of voltage is set at the AC source APS-9501. The heat, released in the liquid, causes it to warm up, and rotation distributes the temperature so that it acquires the maximum value on the cylinder axis. The cuvette rotates at a constant speed until a steady-state temperature distribution is established. This process is monitored according to sensor T_1_ и T_2_ readings and takes from one to three hours depending on the working solution and the power of heat release. Further, an experiment is conducted depending on the problem to be solved.

**Fig. 4:**
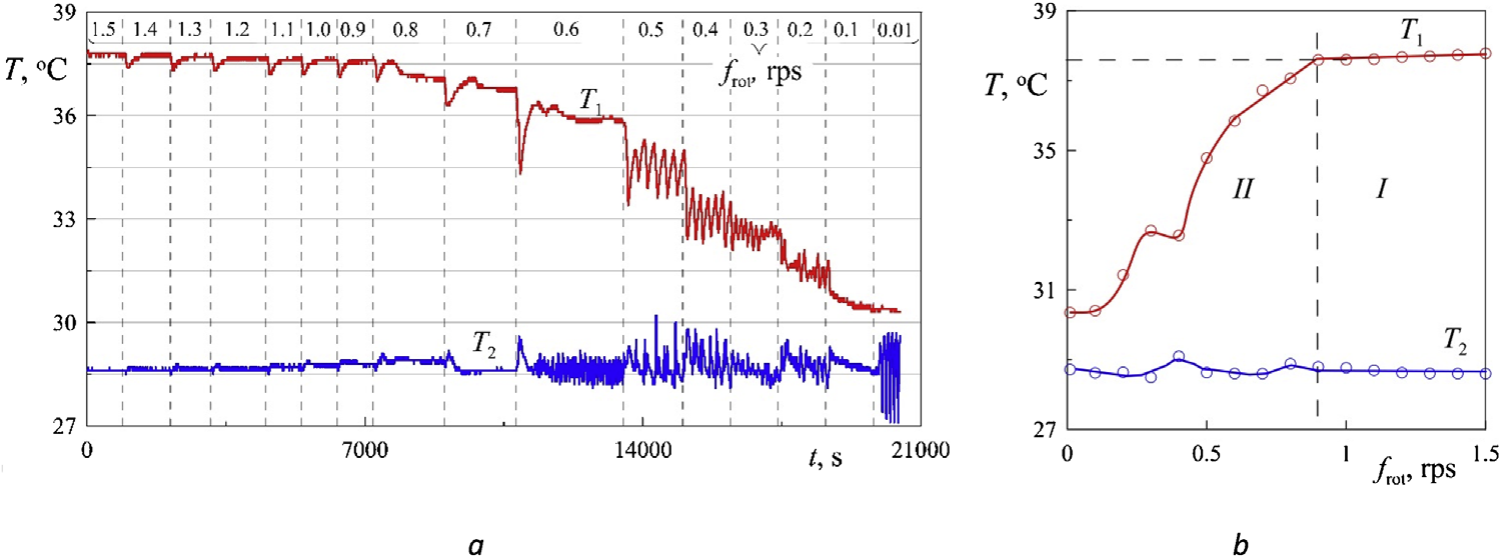
Temperature recording from the integral resistance thermometers (a) and the dependence of the time averaged temperature on the velocity of the cavity rotation (b); power density is 0.087 W/cm^3^.

One of the problem is to study the convection of a fluid in a rotating horizontal cavity in the absence of vibrations. In such a formulation, the field of gravity rotates in the cavity reference system and acts as an oscillating force field (analogous to circular vibrations). Fig. 4(a) shows an example of data recording from temperature sensors for almost 6 h. In the experiment, the rotational velocity of the cavity decreases stepwise. At a certain critical value of the velocity, convective flows leading to a decrease in temperature T_1_ at the axis of the cavity, arise. The step size may vary depending on the task, but in most cases it is 0.1 rps. In particular, in the area of high values of rotational velocity (more than 1.2 rps), where the readings of the sensors practically do not change from step to step, the value of step can be increased to 0.2 rps. In the area of developed convection (from 0.7 rps), on the contrary, it is sometimes necessary to reduce the step to 0.05 rps. At each step, time passes before reaching the stationary mode of convection (10–60 min). With the appearance of temperature fluctuations recorded by the sensors, the time of data collection can be increased to obtain as much information about the period and amplitude of oscillations as possible. Temperature data are taken with frequency 1 s^−1^ (Fig. 4a).

When processing temperature records (Fig. 4), only the results obtained after the establishment of a stationary convection mode are taken into account. Such a sample is necessary to exclude from consideration the transient processes associated with the spin down effect – when the rotational velocity of the cuvette is reduced, the fluid in the cavity volume tends to maintain its own velocity, while the walls carry the boundary layers away [7]. This also applies to the fluid layer near the ends of the cavity. The difference in the velocities of rotation of the fluid layers leads to the appearance of flows that deliver cold fluid from the cylindrical boundary to the central sensor. After some decrease in the temperature on the axis, its increase is again recorded.

After the experiment at each step in the stationary mode of convection, the time average values of temperatures T_1_ and T_2_ are determined and the relative temperature on the axis of the cavity Θ = T_1_–T_2_ is calculated. If there are fluctuations in the sensor readings, an integer number of periods at a given velocity step is selected as the interval for analysis.

The appearance of convective flows in the cavity leads to characteristic fractures on the curves of the dependence of temperature on the velocity of rotation f_rot_ (Fig. 4b). Threshold velocity and the corresponding temperatures T_1_, T_2_ and Θ are determined from graphs according to this fractures (boundary between regions I and II).

When calculating the dimensionless complexes, the temperature dependence of the thermophysical parameters of liquids is taken into account. Values such as density, viscosity, coefficient of volume expansion and thermal conductivity at each experiment step are calculated for the average temperature T = (T_1_ + T_2_) /2 in the cylinder.

A systematic study of the excitation thresholds for convection of a heat-generating fluid in a rotating horizontal cylinder is given in [2,8]. The case of a cylindrical layer is considered in [3]. The experimental results are consistent with the theory of the effect built in [4,9].

Another problem is to study thermal convection in rotating cavities under the action of transverse vibrations. In experiments, an area of high cavity rotational velocity (2–4 rps) is considered. In this case, the mechanism for the excitation of convection described above does not manifest itself. This is due to the significant stabilizing effect of the centrifugal force of inertia.

**Fig. 5:**
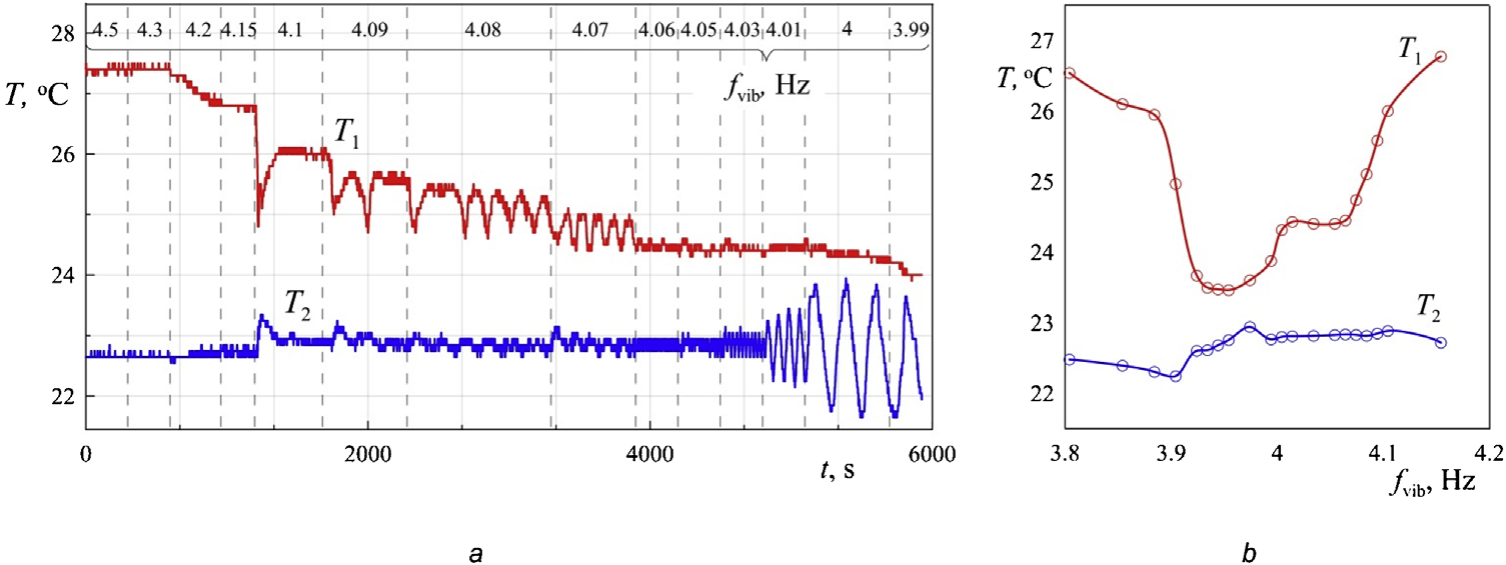
Temperature recording from the integral resistance thermometers (a) and the dependence of the time average temperature on the frequency of vibrations (b); power density is 0.027 W/cm^3^, the amplitude of vibrations b =24.57 mm, the rotational velocity f_rot_ = 4 rps.

Before the beginning of the experiment, the cuvette is brought into relatively fast rotation (2, 3, or 4 rps), heating is turned on. The experiment begins after a steady-state temperature distribution is established in the cuvette. First, the mechanical vibrator sets the vibrations with a fixed amplitude and a frequency exceeding the rotational frequency. Then the frequency of oscillations decreases stepwise. The reaction of the liquid to vibrations is monitored by the readings of the temperature sensors at each step in real time (Fig. 5). The transition to the next frequency is carried out only after the stationary mode of convection has been established in the cuvette (at least 15 min). A series of experiments are carried out at different amplitudes of vibrations.When the frequency of vibrations is significantly different from the frequency of rotation, as in the absence of vibrations, the temperature recorded by the sensors T_1_ and T_2_, remains constant (Fig. 5). Changes of T_1_ and T_2_ related only to the accuracy of the meter 0.1 °C. Temperature decrease in the center of the cavity T_1_ is observed in the case of close values f_rot_ and f_vib_. The detected effect has an applied potential and is associated with the creation of effective tools for the intensification of heat and mass transfer. The systematic studies are carried out in [5].

### Features of the research

#### The effect of the presence of copper sulphate on the parameters of liquid

**Fig. 6:**
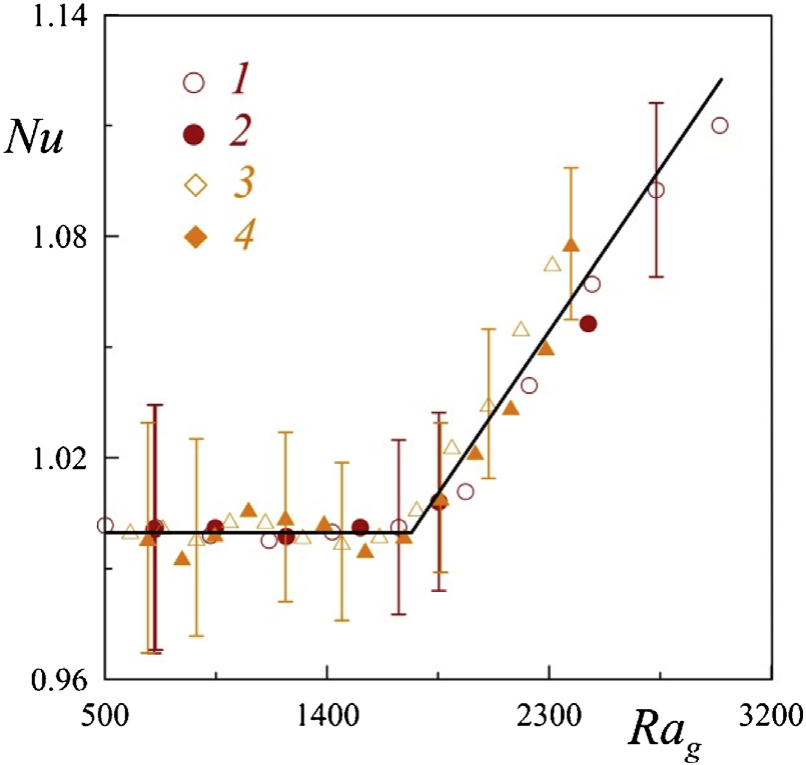
The dependence of the Nusselt number Nu from the gravitational Rayleigh number; water (1), water with copper sulphate (2), water-glycerol solution with mass fraction of glycerol equals 25% (3), water-glycerol solution with copper sulphate (4).

To study the effect of copper sulphate added to the working fluid solution to increase the electrical conductivity, the additional experiments are curried. The excitation threshold of gravitational convection is determined in a thin plane horizontal layer of liquid heated from below [1]. To reproduce the classical experiment, the test liquid located between two flat aluminum heat exchangers. The temperature of the layer boundaries is set by liquid thermostats that pump water in the internal channels of the heat exchangers. This ensures the condition of isothermal layer boundaries, which greatly facilitates thermal measurements and allows the researcher to record the change of modes of convection accurately. With an increase in the temperature difference between the boundaries of the layer, the heat-conducting regime is replaced by convective fluid motion. This change is recorded by a break on the curves of heat transfer (Fig. 6). The critical value of the gravitational Rayleigh number $Ra_{g}=\text{gβ}\Theta h^{3}/\text{νχ}$ for these conditions equals 1708 [1]. Here β, *ν*, and χ are the coefficients of volume expansion, kinematic viscosity and thermal conductivity respectively. The thickness of the plane layer used in the experiments is h =2.3 mm. The heat flux through the layer is characterized by a temperature difference on a plastic gasket 2 mm thick, installed in the massive of the hot (lower) heat exchanger along the entire plane. Measurement of the temperature difference between the boundaries of the layer and the temperature difference on the thermal resistance is carried out by differential thermocouples. Resistance thermometers determine the temperature of the heat exchangers.

The mass fraction of copper sulphate in the experiments is 5%. The change of Ra_g_ in experiments is achieve by stepwise changing the temperature of the hot (lower) heat exchanger, and thereby changing Θ. The temperature of the upper heat exchanger does not change and equals 19.0 °C. The heat transfer characteristic is the Nusselt number Nu defined as the ratio of the heat flux through the layer to the heat flux in the absence of convection in the same experiment. The heat transfer crisis is clearly visible on the dependencies of the parameter Nu on Ra_g_. The results of the experiments are in satisfactory agreement with each other (Fig. 6), and the critical value of the Rayleigh number is reproduced to an accuracy of 3%. This indicates a negligible effect of the presence of copper sulphate on the thermophysical properties of the liquid.

#### Heat loss through the end walls of the cavity

Due to the design features of the cuvette (one of the ends is washed by the coolant together with the sidewalls) and the relatively high thermal conductivity of the material of the flange, the temperature of liquid near the end walls is lower than that far from the flanges. The study of the longitudinal temperature distribution at rapid rotation is performed in experiments with thermocouples, which are installed at the equal distances along the axis of rotation. For this, hot junctions of four differential copper-constantan thermocouples are placed in a glass capillary with a diameter of 2.5 mm. The first junction is installed in a copper electrode plane, the last one – in the center of the cylinder. The cold junctions of all thermocouples are combined and brought to the cylindrical boundary of the internal cavity. To reduce the inertia of thermal measurements, non-conducting oil is poured into the capillary with sensors.

**Fig. 7:**
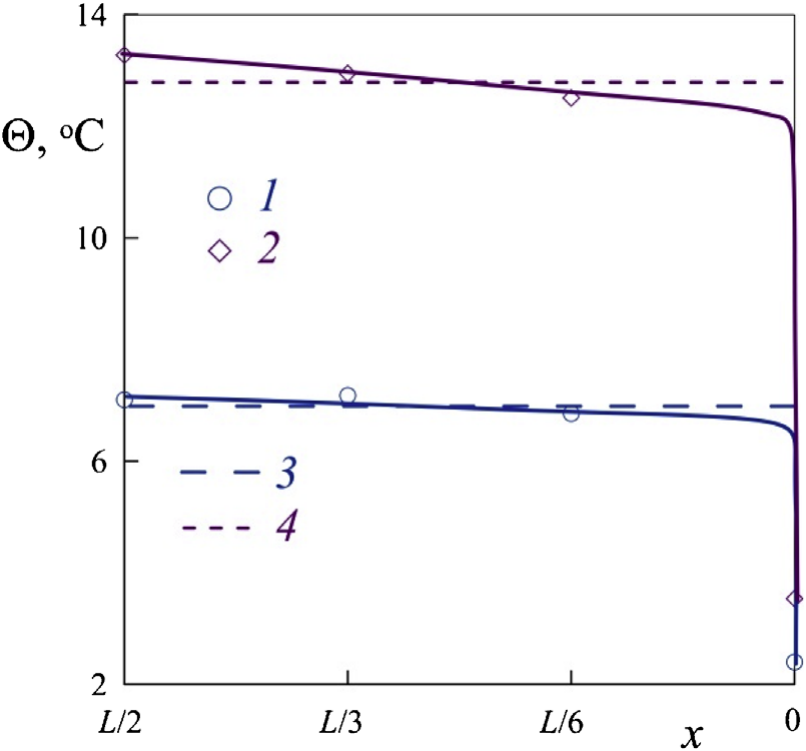
The dependence of temperature Θ at the cavity axis on the longitudinal coordinate x; water; power density is 0.057 (1) and 0.109 (2) W/cm^3^.

Fig. 7 shows the longitudinal temperature profile obtained in experiments with thermocouples. The longitudinal x coordinate is expressed by the length of the cuvette. The reading of the x coordinate starts from the right end of the cavity. The values of x=0, L/6, L/3, L/2 correspond to the positions of the hot junctions of the thermocouples. The temperature Θ at the center and at the end of the cuvette differs by 3–4 times. Solid lines drawn along points 1 and 2 mark the longitudinal temperature profile. The profile is calculated using the integral value of temperature Θ, measured for the same values of power density (dotted lines 3 and 4, respectively). The decrease in temperature along the axis of rotation is not linear: 90% of the decline occurs at a thin layer near the end of the cuvette, while in the other regions the longitudinal temperature gradient is nearly absent.
